# Testing Proximity of Genomic Regions to Transcription Start Sites and Enhancers Complements Gene Set Enrichment Testing

**DOI:** 10.3389/fgene.2020.00199

**Published:** 2020-03-06

**Authors:** Christopher Lee, Kai Wang, Tingting Qin, Maureen A. Sartor

**Affiliations:** ^1^Department of Computational Medicine and Bioinformatics, School of Medicine, University of Michigan, Ann Arbor, MI, United States; ^2^Department of Biostatistics, School of Public Health, University of Michigan, Ann Arbor, MI, United States

**Keywords:** gene set enrichment test, ChIP-seq data analysis, non-parametric test, pathway analysis, genomic regions

## Abstract

Large sets of genomic regions are generated by the initial analysis of various genome-wide sequencing data, such as ChIP-seq and ATAC-seq experiments. Gene set enrichment (GSE) methods are commonly employed to determine the pathways associated with them. Given the pathways and other gene sets (e.g., GO terms) of significance, it is of great interest to know the extent to which each is driven by binding near transcription start sites (TSS) or near enhancers. Currently, no tool performs such an analysis. Here, we present a method that addresses this question to complement GSE methods for genomic regions. Specifically, the new method tests whether the genomic regions in a gene set are significantly closer to a TSS (or to an enhancer) than expected by chance given the total list of genomic regions, using a non-parametric test. Combining the results from a GSE test with our novel method provides additional information regarding the mode of regulation of each pathway, and additional evidence that the pathway is truly enriched. We illustrate our new method with a large set of ENCODE ChIP-seq data, using the *chipenrich* Bioconductor package. The results show that our method is a powerful complementary approach to help researchers interpret large sets of genomic regions.

## Introduction

Cell development and differentiation depend on complex gene expression patterns which are precisely and spatiotemporally controlled. The complex process of gene regulation involves many different mechanisms, including regulation of transcription (Berger, 2007; Deaton and Bird, 2011), post-transcriptional regulation (Roundtree et al., 2017), and regulation of translation (Sonenberg and Hinnebusch, 2009). Transcription is the first step to decode the genetic information from DNA to functional elements, and this process is regulated by many *cis*-regulatory elements across the genome (Wittkopp and Kalay, 2011). *Cis*-regulatory elements include promoters, enhancers, silencers, and insulators, with promoters and enhancers being two important ones that can initiate transcription and are the most well-studied (Andersson, 2015). Both promoters and enhancers are regions of DNA sequences that typically are a few hundred base pairs in length (Nguyen et al., 2016). Promoters are usually located immediately upstream of the transcription start sites (TSSs) on the 5′ end of target genes (Sanyal et al., 2012) and recruit transcription factors (TFs) and RNA polymerase II (RNAPII) to instruct the direction and initiation of transcription (Schoenfelder and Fraser, 2019). Conversely, enhancers can be located upstream, downstream, or in the intron of the target gene or another unrelated gene (Shlyueva et al., 2014) and bound by TFs and cofactors to activate or increase the transcription rate of their target genes (Li et al., 2016). The protein sequences and regulatory motifs of many TFs are well conserved across living organisms, indicating that genome-wide gene regulatory mechanisms have important conserved properties (Lambert et al., 2018). However, some TFs such as ESR1 bind to different sets of target genes in a cell type specific manner (Gertz et al., 2012), resulting in complex and dynamic TF regulatory programs. Thus, deciphering the rules of TF binding events is a key step to understanding gene expression patterns and associated biological pathways.

A diverse collection of sequence-based approaches exist to probe the gene regulome (Pinsach-Abuin et al., 2016). For instance, ChIP-seq can provide genome-wide information about gene regulation for specific TFs or chromatin marks by identifying thousands of genomic regions (i.e., peaks, which we will refer to for simplicity) across the genome (Schmidt et al., 2009). ATAC-seq and copy number variation (CNV) sequencing are also popular for studying genome-wide regulation (Xie and Tammi, 2009; Buenrostro et al., 2015). Through the aforementioned sequencing data, we can identify significant peaks that were bound by a particular TF or modified chromatin mark (ChIP-seq), open chromatin regions (ATAC-seq), or regions with a CNV. We can further infer their underlying regulatory functions by associating the identified regions with target genes, whether predicted or verified. Since biological processes involve many genes and pathways, gene-centered analysis on regulome data may not be as informative as Gene Set Enrichment (GSE) testing (Subramanian et al., 2005).

Most GSE methods were developed for gene expression data, do not adjust for the varying lengths of genes or regulatory space between them, and thus are not generally appropriate for GSE testing with large sets of peaks. However, several GSE methods have been developed to specifically test sets of peaks, including GREAT (McLean et al., 2010), ChIP-Enrich (Welch et al., 2014), Broad-Enrich (Cavalcante et al., 2014), and Poly-Enrich (Lee et al., 2018). Among these, Poly-Enrich is the only method that counts genomic regions (which we will refer to as peaks for simplicity) for each gene, adjusts for the varying lengths of genes and regulatory space between them, and provides a flexible approach with the ability to assign weights to peaks.

Current methods for GSE testing of peaks focus mainly on the relationship between peaks and TSSs (promoters). However, although some TFs [e.g., E2F1 (Ertosun et al., 2016)] preferentially bind to promoters, others [e.g., FOXA1 (Pristera et al., 2015)] tend to bind enhancers, while still other TFs bind to both enhancers and promoters depending on context (e.g., master regulators, such as Serum response factor). Therefore, it is of great interest to know the patterns of TF binding with respect to promoters and enhancers of the target genes and pathways. Although GREAT (McLean et al., 2010), ChIP-Enrich (Welch et al., 2014), and Poly-Enrich (Lee et al., 2018) incorporate distal binding events in their GSE testing, no method has been established for answering the question of whether a TF is binding closer to TSSs, near enhancers, both, or neither for a specific gene set.

Other methods such as ChIPseeker (Yu et al., 2015) and Seq2pathway (Wang et al., 2015) also perform GSE testing for genomic regions. Different from previous GSE testing methods that assign peaks to nearest TSS (NTSS), ChIPseeker applies a max distance cutoff for assigning peaks to genes. Seq2pathway incorporates the significance of each genomic region and both coding and non-coding regions in GSE testing. Methods such as Cistrome-GO (Li et al., 2019) and TREG (Chen et al., 2013) incorporate the distance between ChIP-seq peaks and a gene’s TSS into the GSE testing itself. Cistrome-GO integrates the peak distance to TSS and the peak number together to estimate the gene regulation potential. TREG collects the peak distances to a gene’s TSS within a 2Mb window around each TSS into the GSE test. However, since these methods embed the information about binding proximity to a TSS within the test itself, it is difficult for the user to interpret the results with respect to this information, or separate the effect of proximity from that of enrichment. A recently published, unique tool called loci2path (Xu et al., 2019) links a set of genomic regions to key pathways by testing for enrichment of expression quantitative trait loci (eQTLs) in the genomic regions, including tissue-specific analyses. By using eQTL target genes, loci2path does not rely on assigning genomic regions to the nearest gene, and thus it is a complementary method to a proximity test. No method, to our knowledge, incorporates enhancer proximity.

Here, we propose a new method, Proximity Regulation (ProxReg) to address this shortcoming of current methods. By measuring the distance between each peak and the closest TSS (or enhancer) and then performing a modified two-sided Wilcoxon rank-sum test, we test whether the peaks in a gene set are significantly closer to TSSs or enhancers than expected by chance. Our method, in combination with a GSE test, is able to provide additional evidence that a pathway is truly enriched and information on the regulatory mechanism for that enrichment. After validating the Type I error rate of our method, we test ProxReg by applying it, in combination with Poly-Enrich (implemented in the *chipenrich* Bioconductor package) to 90 ENCODE ChIP-seq datasets (Sloan et al., 2015), including 35 TFs. In many cases, this led to a significant improvement in the ability to pinpoint the known biological processes in which a TF functions. In summary, we show the power and benefits of ProxReg, which is available in five species (fruit fly, zebrafish, mouse, rat, and human) for promoters and in human for enhancers, to complement GSE testing of large sets of peaks.

## Materials and Methods

### Datasets Used

We used a total of 90 human ChIP-seq datasets from the Encyclopedia of DNA Elements (ENCODE) at University of California, Santa Cruz (ENCODE Project Consortium, 2004; Qu and Fang, 2013; Sloan et al., 2015) that consists of 35 TFs over the three Tier 1 cell lines [embryonic stem cells (H1-hESC), B-Lymphocyte (GM12878), and myelogenous leukemia cell (K562)] (Supplementary Table 1).

Gene sets tested were Gene Ontology: Biological Processes (GO BP) from *GO.db* Bioconductor package version 3.4.2 (The Gene Ontology Consortium, 2018). We filtered gene sets to only use those with more than 15 and less than 2000 genes, as small gene sets have very little statistical power and large gene sets tend to be too vague to have meaningful biological interpretation. This resulted in 5159 GO BP gene sets.

### Measuring Peak Distances to Nearest Transcription Start Site or Enhancer Midpoint

Each peak’s “regulatory proximity” was defined as the distance, in base pairs, between the peak’s midpoint and either the closest TSS or the midpoint of the closest enhancer region. Human gene TSS locations were obtained from the *chipenrich* package, which for hg19 version 3.5.0 are from Bioconductor packages *TxDb.Hsapiens.UCSC.hg19.knowngene* version 3.2.2 (Carlson and Maintainer, 2015) and *org.Hs.eg.db* version 3.5.0 (Carlson, 2018). Enhancer regions were defined by the union of DNase hypersensitive sites (DNase DHSs) found in at least two of the 125 cell and tissue types processed by ENCODE (Thurman et al., 2012) and distal and non-promoter DHS within 500 kb of the correlated promoter DHSs from 32 cell types (Thurman et al., 2012). The minimum of two cell types was used to reduce false positives. Unions were calculated using the *expand_and_resect2* function in the *granges* R package with min.gapwidth = 0, and distal and non-promoter elements were defined as those >5 kb from a TSS. That is, we removed only the portion of an enhancer that was <5 kb from a TSS. This resulted in a total set of 1,616,520 regions >5 kb from a TSS composed of enhancers, silencers, and insulators, although for simplicity we refer to the total set as enhancers. Finally, all peaks are then assigned to the gene with the NTSS.

### ProxReg Step 1: Normalizing for Gene Locus Length and Average Distance to Enhancer

Identical to our previous work, we define a gene’s locus length (in bps) as the length of the region on the genome such that a peak binding in the region is assigned to that target gene (Cavalcante et al., 2014; Welch et al., 2014). As genes with larger locus lengths (i.e., longer distances to neighboring genes) are more likely to have peaks binding farther away from the gene’s TSS, gene locus length is associated with average peak distance to TSS, and thus gene locus length is a potential confounding variable. To empirically normalize for gene locus length, we used the combined set of peaks from all 90 ENCODE ChIP-seq peak datasets and computed a cubic smoothing spline for log locus length (*x*-axis) vs. log peak distances (*y*-axis) using the *gam* function in the *mgcv* package. The spline provides the expected, global average binding distance for each gene, which we then used to obtain the normalized adjusted binding distance as:

$$D_{t\,s\,s}^{a\,d\,j}=\log D_{t\,s\,s}-\log D_{s\,p\,l\,i\,n\,e}$$

Thus, peaks that are closer to a TSS than expected based on the spline fit will contribute to significant promoter proximity for a gene set.

Similar to how a gene with a longer locus length tends to have peaks farther from its TSS, gene loci with farther spaced enhancers tend to have peaks farther from them. More specifically, the distance to an enhancer region is associated with how far apart a gene’s enhancers are spread, which is dependent on both the gene locus length and the number and distribution of enhancers associated with the locus region. Therefore, the average (or expected) enhancer density for each gene is a potentially confounding variable. To normalize for this, we first determined every gene’s empirical average distance to an enhancer with our list of 90 ENCODE ChIP-seq datasets, and then calculated each peak’s distance to the nearest enhancer, and finally averaged this distance for each individual gene. As these 90 experiments do not cover every gene, if a dataset happens to have a peak assigned to a gene not covered, the average distance to enhancer will be set as the predicted mean of a linear estimation using the log gene locus length of the known genes. Similar to the locus length normalization, we have the adjusted enhancer distance:

$$D_{e\,n\,h}^{a\,d\,j}=\log D_{e\,n\,h}-\log A\,v\,g\,D_{e\,n\,h}$$

Thus, peaks closer to an enhancer than expected by chance will contribute to significant enhancer proximity for a gene set.

### ProxReg Step 2: Testing for Proximal Regulatory Binding

For a gene set of interest, the peaks assigned to genes in the gene set are placed in one group while all other peaks assigned to other genes, called the background genes, are placed in another. We let any gene that has the potential of a peak being assigned to it and annotated in the gene set database to be a background gene, which is equivalent to the procedure of gene expression tools such as DAVID (Da Wei Huang et al., 2007). The goal is to test whether the peaks in the gene set are significantly closer to TSSs (or enhancers) than expected by chance, given the adjusted distances described above. We use a two-sided Wilcoxon rank-sum test, with positive values denoting the distances in the gene set are *smaller* than those not in the gene set, to test if peaks in the gene set tend to be closer or farther from regulatory regions than those not in the gene set. To account for multiple testing, we use the Benjamini–Hochberg method to calculate FDR values (Benjamini and Hochberg, 1995).

### Gene Set Enrichment Testing Using Poly-Enrich

We tested all 90 ENCODE datasets using the *polyenrich* method in the *chipenrich* Bioconductor package, using the “nearest_tss” gene locus definition and GO biological processes for the gene sets. Poly-Enrich performs GSE on sets of peaks by testing if the number of peaks regulating a gene set is greater or less than that not in the gene set, taking into account the number of peaks assigned to each gene (Lee et al., 2018). The statistical model uses a negative binomial *glm* with an adjustment for gene locus length. Significantly enriched gene sets have more peaks, while depleted ones have fewer.

### Permutations to Assess Type I Error Rate

To test Type I error rate of the ProxReg method, we simulated a null set of peak distances (i.e., with no gene sets having significant proximal binding) in three ways: (1) by reassigning every peak to a random gene, where all genes are equally likely to be assigned (*Unif*). (2) To test for correct normalization of gene locus length, we randomized peaks to a gene as above, except genes were first binned with other genes of similar locus length as defined by their TSSs. Specifically, we ranked genes by locus length, binned them into sets of 100 genes, and then reassigned every peak to a random gene within the same bin (*ByLocusLength*). (3) To test the normalization of average distance to enhancers, we ranked genes by expected distance to enhancer by chance, and then binned genes into sets of 100. Again, we then reassigned every peak to a random gene within the same bin (*ByAvgDEnh*). We performed 10 randomizations per ChIP-seq experiment and chose α-levels of 0.05 and 0.001 to test for a controlled Type I error rate.

### Simulations to Estimate Power

We simulated significant proximal gene sets by starting from a null set of peaks using the *ByLocusLength* permutation strategy. We then added peaks near the TSSs of genes from a gene set, with the choice of a small (471 genes) or a large (1717 genes) gene set. The number of peaks added was equal to 0.01, 0.05, or 0.1% of the total number of starting peaks (4839) in the null set. The distance was chosen from an exponential distribution with mean *d*_0_, and an equal chance for upstream or downstream. We chose values of 100, 500, 1000 for *d*_0_ to simulate scenarios of closer and farther binding. For each scenario, 200 simulated gene sets were ran.

### Clustering for TF Regulatory Patterns

To investigate the regulatory patterns among all 90 ENCODE ChIP-seq data sets, we performed clustering to classify them. We first applied a *p*-value cut off (<0.001) for both ProxReg (promoter and enhancer) results and Poly-Enrich results. We counted the numbers of points (GO BP terms) in each of four regions, defined by the different colored regions shown in Figure 3, for both promoter and enhancer results in all 90 data sets. Then, a hierarchical clustering heat map was generated based on the log 2 value of counts from each region. The Euclidean distance metric was used with Ward’s minimum variance method for clustering. In addition, we also calculated the Pearson correlation between ProxReg promoter results and enhancer results. Since we propose our method as a complementary method for GSE testing, only signed negative log *p*-values of significant GO terms (FDR < 0.05) from Poly-Enrich were used for correlation calculations.

### Test for the Ability of ProxReg to Reduce False Positives From GSE Results

To test the ability of our method to reduce false positives from GSE results, we compared the results of ProxReg and Poly-Enrich together to Poly-Enrich alone, using gene sets for each TF that the TF is likely to regulate. Since no gold standard is available for this, we used the GO BP terms that our 35 TFs were assigned to in the human annotation Bioconductor package *org.Hs.eg.db* (Carlson, 2018). Motivation for this derives from the fact that TFs do not regulate random sets of genes, but rather a well-coordinated set of genes in order to fulfill a cellular biological goal. Indeed, it’s been shown that genes in a GO biological process term tend to be regulated by a common TF (Allocco et al., 2004; Qian et al., 2005; Roider et al., 2008; O’Connor et al., 2016). The cellular biological goal is precisely what GO biological process terms aim to describe, as it is defined as “The larger processes, or ‘biological programs’ accomplished by multiple molecular activities” (The Gene Ontology Consortium, 2004), which for TFs in DNA binding. Based on these two facts, the TFs that are assigned to a GO biological process term relate closely to this biological process, and since the function of TFs is to regulate genes, it follows logically that TFs tend to regulate genes in the biological processes to which they belong. As an example, the NCBI Gene website, an authoritative source for the properties of genes, states in the main summary of E2F family genes that “the E2F family plays a crucial role in the control of cell cycle”. This family includes members E2F1, E2F2, E2F3a, E2F3b, E2F4, E2F5, E2F6, E2F7, and E2F8. In each case, we can also find at NCBI Gene that these TFs are assigned to the GO BP terms related to cell cycle. To further validate our approach, we tested whether the TFs actually do tend to target the promoters of genes in their assigned GO terms. Indeed, we found a strong overall trend to targeting more genes in the assigned GO terms versus the non-assigned GO terms (Supplementary Figure 1 and Supplementary Table 2). Although TFs may not regulate all of their target gene sets in every cell type, we conclude that the degree of overlap between a method’s predictions and a TF’s assigned GO BP terms represents a useful benchmarking tool.

To assess this, we first counted all significantly enriched gene sets from Poly-Enrich for all 90 ENCODE ChIP-seq data sets and found their overlap with the GO BP terms each TF was assigned to in *org.Hs.eg.db*. These GO terms were used to count significant GO terms from ProxReg promoter and enhancer results. Fisher’s exact test was used to determine whether ProxReg further enriched the resulting GO terms to those assigned to by the TF, beyond what GSE testing accomplished. We used datasets for TFs that are assigned to at least five GO BP terms that were also significant with GSE testing alone. Fisher’s exact test results demonstrated whether ProxReg was able to increase the odds ratio of identifying GO BP terms assigned to the TF, compared to GSE testing alone.

### Website Implementation and Bioconductor Availability

Proximity Regulation is available in the *chipenrich* Bioconductor package with the *proxReg()* function, and at the ChIP-Enrich website^1^, as an additional option following any of our current GSE tests. To run ProxReg, the user uploads a file of peaks, which can be in narrowPeak or BED format. They then select to test for proximity to either NTSS or enhancers. Currently, we have only implemented testing for enhancer proximity in human (hg19 genome), but others will be added as enhancers are sufficiently defined in other species and newer genome versions. Finally, the user selects what gene sets to test from any of our included gene set databases (including KEGG, Panther, MSigDB gene sets, and several others; details in *chipenrich* package and on website), or a user-generated set. An example of the *proxReg()* function outputs four files:

***Opts***: the options that the user input into the function.
***Peaks***: a peak-level summary showing the peak-to-gene assignment for each peak, as well as their distances to TSS or enhancer.
***Results***: the results of the proximity tests. Lists the tested gene sets along with their descriptions, the test effect, closer/farther status, *p*-value, and FDR. Also included is the list of Entrez gene IDs with contributing signal for each proximity test.
***Qcplot***: a histogram showing the distribution of peak distances.

All R code for recreating analysis and figures can be found at: https://github.com/sartorlab/proxReg. An example for the use of ProxReg can be found in the *chipenrich* Bioconductor vignette.

## Results

### Overview of ProxReg Method

**FIGURE 1:**
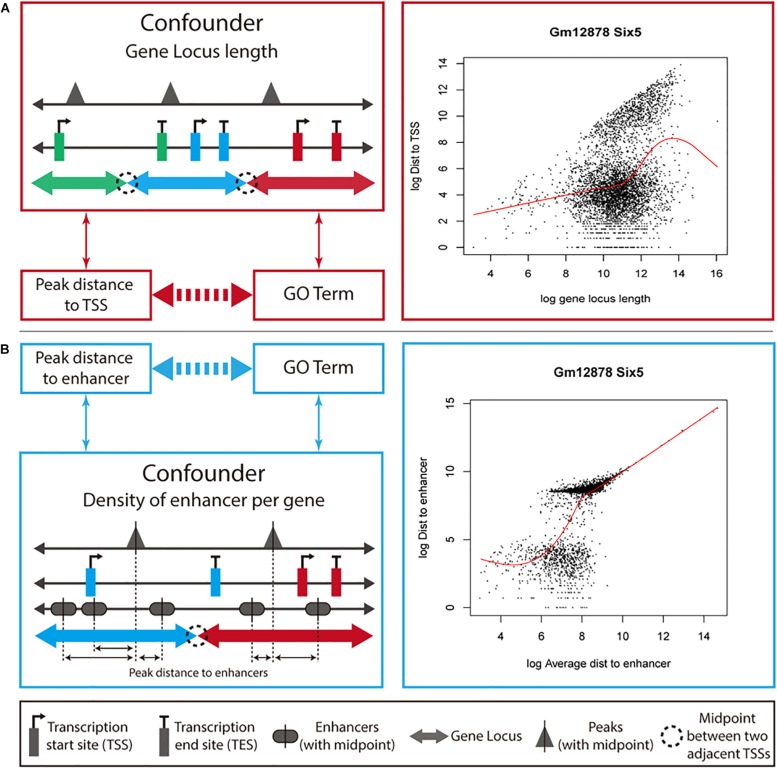
Overview of how ProxReg adjusts for confounding variables. We describe the ProxReg adjustments in two parts. **(A)** When testing proximity to TSSs, we normalize the peak distances to TSSs according to their relationship with gene locus lengths. **(B)** When testing proximity to enhancer, we normalize the peak distances to enhancers according to their relationship with enhancer density, modeled by the average distance of any peak to an enhancer. In both cases, we avoid a potential confounding effect, as shown by the arrows between variables on the left-hand side.

We developed a new method, ProxReg, to test the proximity of peaks to TSSs or enhancers in a gene set of interest. The motivation for our new method is illustrated in Figure 1. The goal is to test whether the enrichment of a GO term or pathway is driven by regulation via promoters or distal regions (i.e., enhancers). To accomplish this, we firstly measure the distances from the midpoints of the peaks to the nearest regulatory regions (either TSSs or enhancers), and assign each peak to its target gene according to the gene with the NTSS (Welch et al., 2014). Specifically, for each gene we defined its gene locus to be the region between the upstream and downstream midpoints of its TSS and the neighboring gene’s TSSs. However, one cannot simply directly test whether the distances are smaller within a gene set versus other genes, due to potentially confounding variables that first need to be taken into account. Since a gene locus with a large length was observed to have farther peaks from its TSS on average (Figure 1A), we first normalize for the gene locus length before testing the proximity to TSSs (see section “Materials and Methods”). For enhancers, we observed that the distance to an enhancer was dependent on the average distance from each enhancer to peaks in a gene locus (Figure 1B). Thus, we normalized the raw peak to enhancer distances using the average enhancer density for each gene. Finally, a two-sided Wilcoxon rank sum test was used for testing the proximity of peaks in a gene set to TSSs (or enhancers) compared to peaks outside the gene set. Generally, this test would be performed on all of the enriched gene sets identified by a GSE test, to understand whether the enrichment of each gene set was due to regulatory activity near promoters or enhancers.

### Recommended Workflow for ProxReg

**FIGURE 2:**
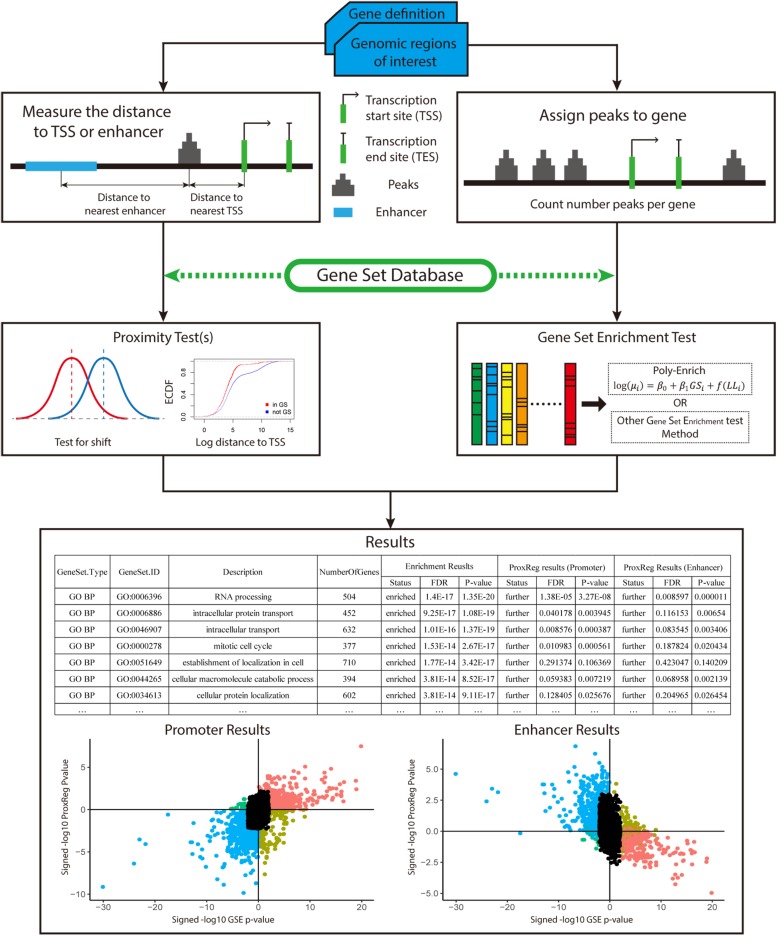
Overview of how of ProxReg fits in with the overall workflow of gene set enrichment testing with genomic regions. The peak distances to TSSs or enhancers are calculated for the proximity test. In parallel, all peaks are assigned to genes for gene set enrichment testing. The same gene set database is used for both proximity and gene set enrichment testing. Combining the gene set enrichment and proximity tests, the results can be visualized as shown in section “Results.” The left scatter plot is an example of the combination of enrichment and promoter results. The right scatter plot is an example of enhancer results combined with enrichment results. The *x*-axis of these two scatter plots represent the gene set enrichment test result. A larger signed –log *p*-value indicates more enrichment, while negative values indicate depletion. The Y-axis represents the proximity results. Larger signed –log *p*-values indicate GO terms having genomic regions closer to the TSSs or enhancers.

To test our new method, 90 ENCODE ChIP-seq data sets (36 TFs in three Tier 1 cell lines) (ENCODE Project Consortium, 2004; Qu and Fang, 2013; Sloan et al., 2015) were used in this study. The recommended workflow for implementing our new method is summarized in Figure 2. We begin with a gene definition file containing gene locus definitions (provided by our software, or uploaded custom by the user) and a set of peaks of interest (provided by the user). The distance between the midpoint of peaks and NTSSs (or midpoint of enhancers) are measured and adjusted for all background genes. The ProxReg non-parametric test is ran for the chosen gene sets (e.g., GO). In parallel to this, a standard GSE test is performed using the same gene sets. In this article, we applied the *polyenrich* method for the GSE test (Lee et al., 2018), but others may be used. Result files contain the proximity results with test direction (enriched/depleted from GSE, and closer/farther from ProxReg), *p*-values and FDR values. Combined with the *p*-values from GSE, the gene set proximity and enrichment patterns can be easily visualized (Figure 2; see section “Results”).

### Controlled Type 1 Error Rate and Ability to Detect True Positive Results

We validated the Type 1 error rate (rate of false positives) of ProxReg using randomizations of real datasets to simulate null datasets with no significant proximities to TSSs or enhancers. We performed three types of permutations: the “*Unif*” permutation, which takes every peak and reassigns another gene to it with each gene having the same probability, the “*ByLocusLength*” permutation, which tests the effectiveness of the locus length normalization in the distance to TSS test, and the “*ByAvgDEnh*” permutation, which tests the effectiveness of the normalization to average distance to enhancer in the distance to enhancer test (see section “Materials and Methods” for details). For a *p*-value < 0.05 cutoff, we expect a Type I error rate of approximately 5%. For a *p*-value < 0.001 cutoff, we expect a Type I error rate of approximately 0.1%. Results indicate that for each permutation (*Unif* and *ByLocusLength* for TSS proximity tests, and *Unif* and *ByAvgDEnh* for enhancer proximity tests), the Type 1 error rate is reasonably controlled at the expected level (Supplementary Figure 2).

To ensure that our method is able to identify gene sets with true cases of TSS or enhancer proximity, we generated artificial peak datasets starting with a randomized data set using the *ByLocusLength* permutation, and then adding peaks with TSS distances following a specified distribution. We added peaks by varying the number of peaks and the distance of peaks to assess a wide range of scenarios. We also used two gene sets of different sizes (see section “Materials and Methods” for details). We expected the following changes in parameters to increase power: a smaller gene set used (easier to influence average distance), more peaks added, and a smaller average distance. We can see that all three of these scenarios increased power to detect the true positive gene sets as expected (Supplementary Figure 3).

### Integration of GSE and ProxReg Results Reveals Different Regulatory Patterns of TFs

**FIGURE 3:**
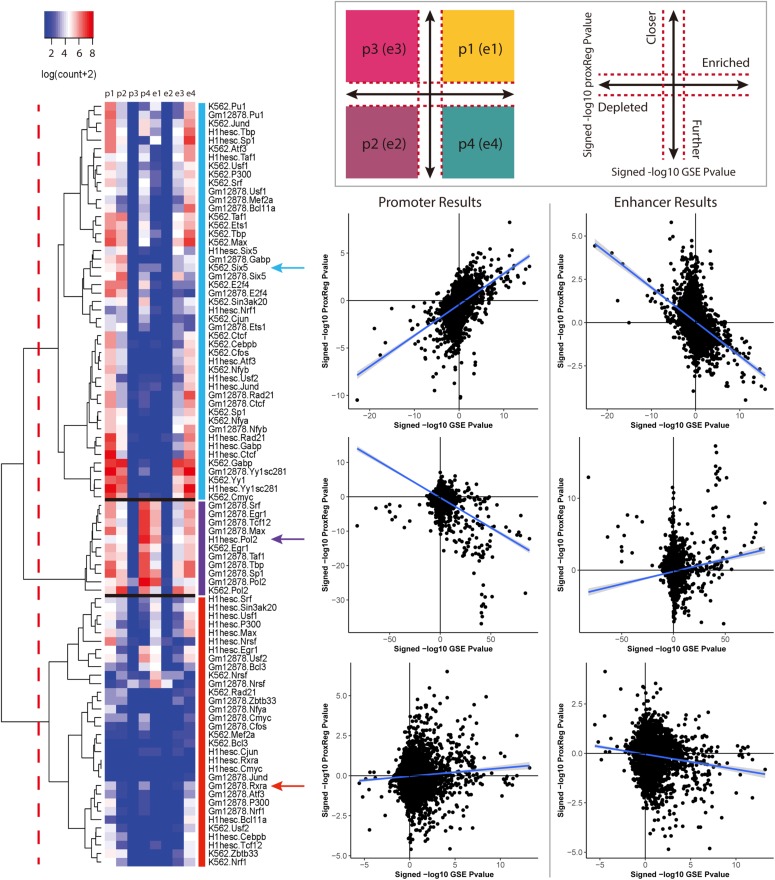
The regulation patterns of the 90 ENCODE ChIP-seq datasets. A *p*-value cutoff (<0.001) was applied to define the four regions as illustrated in the top panel. The cutoffs are represented by the red dash lines. For each data set, the points count of the combination of ProxReg promoter results and Poly-Enrich results are labeled as p1, p2, p3, and p4. Similarly, the combination of enhancer results and Poly-Enrich results are labeled as e1, e2, e3, and e4. Based on our analyses, 47 data sets show a clear positive correlation in promoter results and a clear negative correlation in enhancer results. 32 datasets show no strong correlation in either promoter or enhancer results. The remaining 11 data sets show a clear positive correlation in the enhancer results. For each group, the promoter and enhancer results of one data set are illustrated as an example.

**FIGURE 4:**
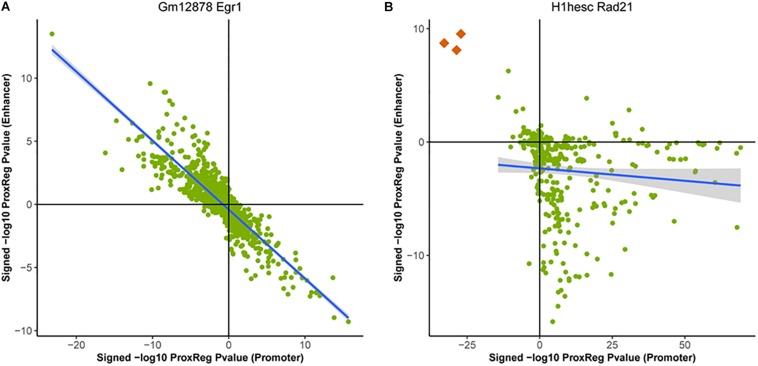
Examples of the correlation between ProxReg promoter *p*-values and enhancer *p*-values. Majority of the 90 ENCODE ChIP-seq data sets show a strong negative correlation as shown in **(A)**. A small portion of these data sets show a pattern as shown in **(B)**. The three orange dots in **(B)** are GO terms related detection of chemical stimulus (GO:0050907, GO:0009593, and GO:0050911).

We clustered the 90 ENCODE ChIP-seq datasets into three groups based on the hierarchical clustering heat map illustrated in Figure 3. The first and largest group (47 datasets) is characterized by a strong positive correlation between GSE and promoter (TSS) ProxReg signed significance levels, and a strong negative correlation between GSE and enhancer ProxReg signed significance levels, indicating that the majority of enriched gene sets are due to binding near TSSs (Figure 3 blue cluster; many genes in regions p1, p2, e3, and e4). TFs like SIX5 (SIX homeobox 5), SP1 (Specificity Protein 1^∗^), and GABP (Nuclear Respiratory Factor 2) are included in this group. The second largest group (32 datasets) is more interesting because the datasets consist of some enriched gene sets with significant proximity to promoters, and other enriched gene sets with significant proximity to enhancers (Figure 3 red cluster; genes spread out across mainly p1, p4, e1, and e4). The results for these TFs enable understanding the different regulatory mechanisms used for different biological processes. MEF2A (Myocyte-specific enhancer factor 2A) in K562 cells, a member of this group, was observed to regulate GTPase activity and translational initiation-related GO terms from TSSs, and transmission of nerve impulse and multicellular organismal signaling GO terms from enhancers. Similarly, P300 (Histone acetyltransferase p300), a well-known marker of enhancers, was found to regulate chromatin organization from TSSs, while regulating phosphatidylinositol dephosphorylation and phosphatidylinositol-mediated signaling-related GO terms from enhancers (Fryer et al., 2002; De Luca et al., 2003). The smallest group included only 11 ChIP-seq datasets. This group was characterized mainly by enriched gene sets with many having significant proximity to enhancer regions and/or far from promoters (Figure 3 purple cluster; many genes in p4 and e1). Members of this group included Pol II in all three cell lines and EGR1 in K562 cells and Gm12878 cells, indicative of Pol II binding along entire gene lengths and not just at promoters. In addition, we examined the Pearson correlation between promoter results and enhancer results for all 90 ENCODE ChIP-seq data sets. Eighty-eight of them show a negative correlation between the promoter results and enhancer results (Figure 4). This negative correlation indicates that overall, GO terms are significantly enriched either by the TF binding closer to promoters or closer to enhancers. Among these 90 data sets, most of them (67 out of 88 data sets) show a strong negative correlation as shown in Figure 4A. Several of them have weak correlations as shown in Figure 4B. The two datasets that did not show negative correlations are neuron restrictive silencer factor (NRSF) and CMYC in H1-hESC cells. After removing non-significant GO BP terms from Poly-Enrich results, NRSF data set shows a weak positive correlation based on the remaining GO BP terms and no significant GO BP terms in CMYC data set.

### ProxReg Identifies Known Associations With Promoter and Enhancer Binding, Using SIX5 and NRSF Peaks

**FIGURE 5:**
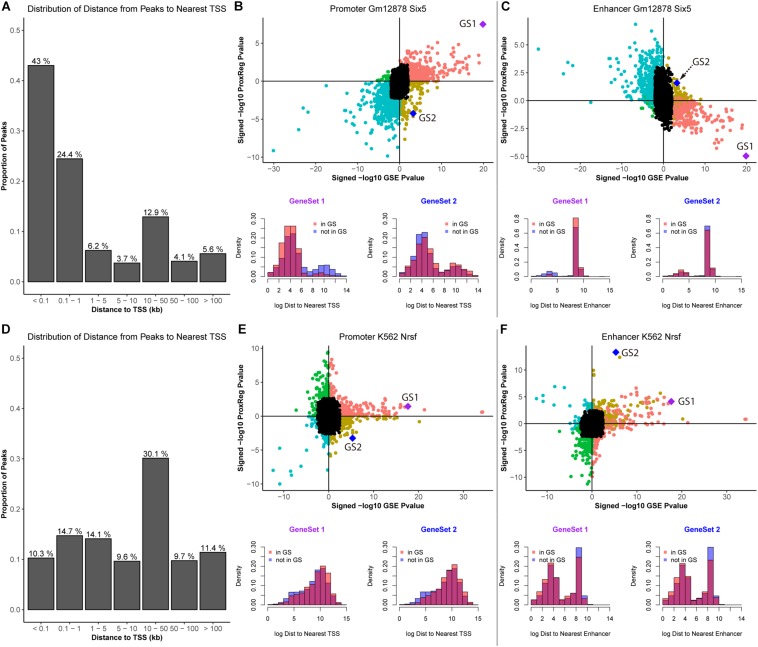
Illustration of ProxReg results. The results of SIX5 in GM12878 cell lines are shown in **(A–C)**. **(A)** The distribution of distances from peaks to nearest TSSs. **(B)** Scatter plot of the combination of enrichment results and promoter results. Two gene sets were selected to show the distance distribution to nearest TSSs for genes in the gene set and not in the gene set. **(C)** Enhancer results combined with the enrichment results. The same gene sets were used in this scatter plot. The distribution of distances to the nearest enhancers of these two gene sets are shown in the bottom of **(C)**. Similar to SIX5 results, **(D–F)** show the results of NRSF in K562 cells. For SIX5, GeneSet 1: RNA processing. GeneSet 2: Positive regulation of nitrogen compound metabolic process. For, NRSF, GeneSet 1: Neuron differentiation. GeneSet 2: System process.

To further illustrate our method, we assess ProxReg results for two TFs known to have a very strong tendency to bind either in proximal promoters or enhancers. We first selected SIX5 in GM12878 cells as an example, which is involved in determination and maintenance of retina formation that proposed binding to promoter regions of related genes (e.g., myogenin and IGFBP5) (Spitz et al., 1998; Sato et al., 2002). The results of SIX5 are shown in Figure 5.

In Figure 5A, we can see that the majority of the ChIP-seq peaks (67.4%) are near TSSs. Through the combination of ProxReg results and Poly-Enrich results, a great majority of gene sets are enriched by the TF binding near TSSs (positive correlation in Figure 5B) instead of near enhancers (negative correlation in Figure 5C). Using two particular GO terms from the scatter plots, we show the distribution of distances from peaks to TSSs or enhancers (bottom part of Figures 4C, 5B). Combining the locations of these two GO terms (GS1 and GS2 in the scatter plots), illustrates how our method is able to provide additional information for interpreting GSE testing results.

We also selected NRSF in the K562 cells as an example. NRSF, also known as RE1-Silencing Transcription factor (REST), is a TF known to silence neuronal genes in non-neuronal cells, it can act as a transcriptional repressor or enhancer of target genes, often regulating from enhancer regions (Schoenherr and Anderson, 1995; Seth and Majzoub, 2001). Almost half of NRSF ChIP-seq peaks (51.2%) are far from TSSs (Figure 5D). A similar strategy was used for illustration of ProxReg with the transcriptional repressor NRSF in K562 cells. Consistent with previous observations that this TF tends to bind to silencers/enhancers instead of promoters, there is a relative strong positive correlation shown in the enhancer scatter plot (Figure 5F) but not for TSSs. Thus the results confirm that most enriched GO terms were enriched due to the TF binding in or near enhancer regions. These results validate our new method, ProxReg, is a powerful tool that can be used as a complementary approach for interpreting GSE test results.

### ProxReg Enriches GSE Findings for Likely True Positives

We assessed whether ProxReg can be used to not only estimate the proximity effects but also help users to remove possible misleading or false positive gene sets from GSE results. To accomplish this, we compared the significantly enriched gene sets to a set of GO biological process (BP) terms from *org.Hs.eg.db* for each TF before versus after taking into account their ProxReg results. The GO BP terms from *org.Hs.eg.db* consists of the TFs and the assigned GO BP terms for the gene that encodes them (see section “Materials and Methods” for more detail).

We used ChIP-seq datasets with at least five significantly enriched GO BP terms in their *org.Hs.eg.db* set (to ensure sufficient power), which resulted in 28 datasets with ProxReg enhancer results and 36 datasets with ProxReg promoter results. We then tested whether requiring a significant ProxReg test resulted in a higher odds ratio of detecting the *TF-assigned* GO BP terms. Of the 28 enhancer dataset results, 18 (64%) had an odds ratio greater than 1. Among these, 11 (61%) of them were significant. Conversely, only two enhancers’ results had an odds ratio significantly less than 1. These two results were from EGR1 and ATF3 in K562 cells. Previous research (Cullen et al., 2010) suggests that EGR1 recognizes and binds to promoter regions of target genes, so it is possible that the GO BP terms from *org.Hs.eg.db* we compared to is incomplete, with previous data mainly being focused on biological processes that EGR1 regulates from promoter regions. A similar case may be true for ATF3.

Among 36 ProxReg promoter results, 25 (69%) had an odds ratio greater than 1. Among these 25 results, 15 (60%) of them were significant. Conversely, only two promoter results were significant with an odds ratio smaller than 1. One of them was PU.1 in K562 cells. A previous study (Heinz et al., 2015) indicated that PU.1 usually binds to a PU-box found on enhancers of target genes, consistent with the ProxReg promoter results of PU.1 peaks having an odds ratio less than 1. Although we only found five significant GO BP terms from our results that are also assigned to PU.1, some other significant GO BP terms that we identified were biologically related to the remaining GO terms assigned to PU.1. For instance, some GO terms assigned to PU.1 were related to response to toxic substances, drugs, and antibiotics, and many immune response-related GO terms were significant. Overall, these results demonstrate that ProxReg can be used as a powerful supplemental method to remove misleading or false positive GSE test results (Supplementary Table 3), and provide additional evidence for novel regulated processes initially identified by GSE testing.

### ProxReg Analysis Identified NRSF Regulatory Pattern Switching in Different Cell Types

**FIGURE 6:**
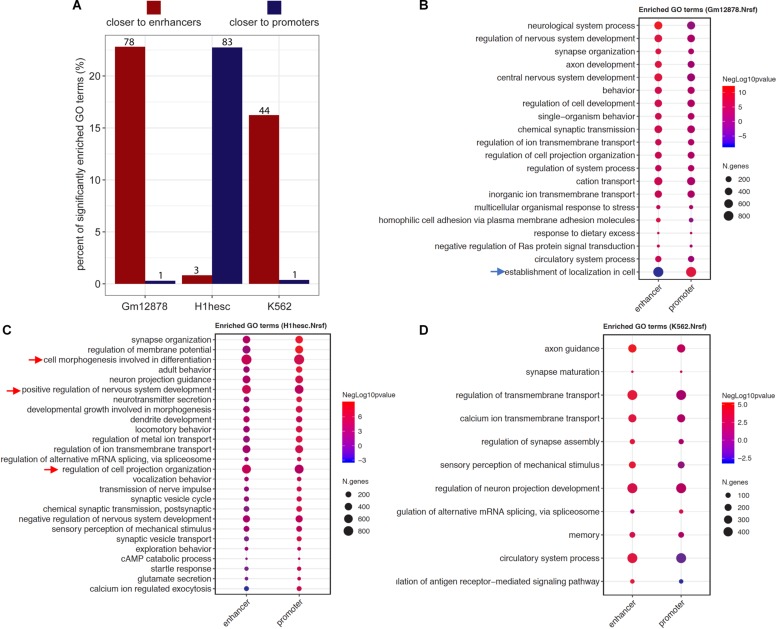
The different regulatory patterns of NRSF in three cell lines. **(A)** The bar plots show the percentage of significantly enriched GO terms that were closer to enhancer (dark red) or promoter (dark blue) in each cell line (x-axis). The numbers of terms were marked on the top of each bar. **(B–D)** The dots represent the ProxReg enhancer or promoter significance levels (signed negative log *p*-values, resulting in positive values for proximal regions, and negative values for more distal regions) of the enriched GO terms in GM12878 **(B)**, H1-hESC **(C)**, and K562 **(D)** cell lines. In a particular cell line, the arrows point to the GO terms closer to promoters (blue arrows) while most of the terms are closer to enhancers, or point to the GO terms closer to enhancers (red arrows) while most of the terms are closer to promoters. For visualization, the redundant GO terms were removed from the list (Koneva et al., 2018).

The ProxReg results can guide and refine the biological interpretation of GSE results by identifying whether each enriched gene set is regulated mainly via binding close to promoters or enhancers. We exemplified this using the findings of NRSF, which was shown to regulate neuron development mostly via binding to enhancers in K562 cells (see details above). To further investigate the regulation patterns of NRSF in different cell lines, we utilized ENCODE NRSF ChIP-seq experiments from three cell types (GM12878, H1-hESC, and K562), and performed and integrated the Poly-Enrich and ProxReg analyses for each cell type. In GM12878, almost all significant GO terms identified by both Poly-Enrich and ProxReg were found to be closer to enhancers, except one GO term “establishment of localization in cell”, which was significantly closer to promoters (FDR = 2.04 × 10^–6^) and farther from enhancers (FDR = 9.60 × 10^–7^) (Figures 6A,B and Supplementary Table 4). Most of them were related to neuron development, including “neurological system process,” “regulation of nervous system development,” and “synapse organization.” In H1-hESC cells, however, NRSF binding sites were significantly enriched in GO terms which were significantly closer to promoters, and mostly related to neuron development and regulation, such as “synapse organization,” “neuron projection guidance,” and “neurotransmitter secretion” (Figures 6A,C and Supplementary Table 5). Less than 1% GO terms were closer to enhancers (“cell morphogenesis involved in differentiation,” “regulation of cell projection organization,” and “positive regulation of nervous system development”). The pattern observed in K562 was similar to that in GM12878: the majority of enriched GO terms were significantly closer to enhancers, and again most of them were related to neuron regulation (e.g., “axon guidance,” “synapse maturation,” and “regulation of synapse assembly”) (Figures 6A,D and Supplementary Table 6), whereas only one was closer to promoters (“regulation of alternative mRNA splicing, via spliceosome”). These findings point to a fundamental shift in the binding patterns of NRSF to regulate neuronal genes during neuron development and organization processes: closer to promoters of genes in H1-hESC, while closer to enhancers in differentiated cells (GM12878 and K562). Taken together, we demonstrate that ProxReg analysis complements the GSE results by distinguishing where a TF binds to regulate genes, which is key to understanding the mechanisms of gene regulation and guiding potential targeted gene therapy. ProxReg is incorporated in the *chipenrich* Bioconductor package and ChIP-Enrich website, and can be used with many additional databases of gene sets.

## Discussion

We introduced a genomic region proximity test called ProxReg that can be used as a complement for GSE tests, and can be used with various types of genomic regions, including ChIP-seq, ATAC-seq, GWAS SNPs, DNA methylation, and repetitive element families. The standard GSE tests for sets of genomic regions (e.g., ChIP-seq peak sets) usually only consider the relationship between the genomic regions and TSSs (McLean et al., 2010). However, it is of great interest to know whether a gene set is significantly enriched through regulatory activity near promoters or enhancers. Our new method, ProxReg, is able to find gene sets with regions that bind significantly closer to (or farther from) either promoters or enhancers. Furthermore, we validated that it has an appropriate Type I error rate, and that the statistical power of the test behaves as expected when varying the relevant variables. ProxReg uses a two-sided Wilcoxon rank-sum test for the proximity test while adjusting for important confounding variables. On its own, it provides insight into particular regulatory patterns. Integrated with GSE testing, it serves as a powerful complementary approach to enhance understanding of regulatory behavior across cell types, time points, disease stages, and more.

When performing pathway analyses with current tools, the method may detect significance from regulation coming from different regions, but the underlying details are often left unknown. Standard GSE tests either do not take proximity to regulatory regions into account, or embed the proximity to TSSs within the test, still ignoring enhancers. In this way, it is difficult to interpret the results without the proximity effects. For example, when GREAT or Poly-Enrich finds a significant gene set from a ChIP-seq experiment, it is known that the gene set is enriched with peaks compared to genes not in the gene set, but we do not know if the peaks reside in promoter or enhancer regions any more than expected by chance. ProxReg is able to further show if the binding sites are closer to (or farther from) TSSs or enhancers, giving more insight into a TF’s binding tendencies. We showed with real world ChIP-seq datasets from ENCODE that ProxReg was able to identify tendencies of TFs known to most often bind in proximal promoter regions (SIX5) (Spitz et al., 1998; Sato et al., 2002) or distal regions (NRSF) (Schoenherr and Anderson, 1995; Seth and Majzoub, 2001). Additionally, significantly enriched gene sets that were not found to be significant by ProxReg may have resulted from distal peaks being misassigned to incorrect target genes.

To illustrate the usefulness of ProxReg, we performed GSE and ProxReg testing on three ChIP-seq datasets of the TF NRSF in embryonic stem cells (H1-hESC) and two differentiated cell lines (K562 and GM12878). We showed how NRSF tends to regulate certain neuronal-related gene sets in differentiated cells by binding closer to enhancer regions, while regulating similar gene sets via binding to promoters in embryonic stem cells. Furthermore, we identified other non-neuronal GO terms that NRSF regulates via binding mainly in promoter (or enhancer) regions. It is interesting to note that the enhancer binding, which is more cell-type specific and generally evolved later than regulation from promoters (Nord et al., 2013; Cai et al., 2019), was identified for the complex neuron development and related terms, while more basic processes such an establishment of location in cell and mRNA splicing, were regulated from closer to TSSs. Only in embryonic stem cells was even the neuronal-related terms regulated via promoters.

Proximity Regulation does have multiple limitations. Currently, we have implemented distance to enhancers for human (hg19 and hg38), and are planning to soon provide support for mouse (mm9 and mm10) (Haeussler et al., 2018). Since the enhancer landscape for other organisms lags the comprehensiveness of that for humans and mice, we currently only offer the promoter proximity test for other species. As other organisms’ enhancer locations become more accurately defined, we plan to add support for more enhancer proximity tests.

An ongoing question is the identity of the targeted genes of enhancers binding events (Rubtsov et al., 2006; Sanyal et al., 2012; Melamed et al., 2016), which remains challenging due to long-range chromosome interactions. By analyzing TFs that tend to bind far from TSSs, we found that there are gene sets that tend to be regulated by TFs binding significantly farther from gene TSSs while also binding closer to enhancer locations. However, ProxReg assumes that each peak is associated with the gene with the NTSS, whereas this is often not true. It has been estimated that 79–95% of TF binding actually regulates a gene interceded by one or more other genes (Van Heyningen and Bickmore, 2013; Aldrup-Macdonald and Sullivan, 2014; de Sotero-Caio et al., 2017). Additionally, we used one general set of enhancer locations across the entire genome, whereas in reality, this method may benefit from allowing different tissues to have different sets of defined enhancer locations. Further research is required to understand how the comprehensiveness of the enhancer database affects the results of ProxReg, as well as of GSE tests. We are currently undergoing research on the differences in enhancer locations and their target genes in relation to GSE testing.

## Data Availability Statement

We used a total of 90 human ChIP-seq datasets from the Encyclopedia of DNA Elements (ENCODE) at University of California, Santa Cruz (details found in Supplementary Table 1).

## Author Contributions

MS conceived of the study. CL, KW, and TQ carried out the analysis. CL, KW, TQ, and MS wrote the manuscript.

## Conflict of Interest

The authors declare that the research was conducted in the absence of any commercial or financial relationships that could be construed as a potential conflict of interest.

## Footnotes

1 http://chip-enrich.med.umich.edu
