# Supplementary material for: Testing Proximity of Genomic Regions to Transcription Start Sites and Enhancers Complements Gene Set Enrichment Testing
Source: Front Genet. 2020 Mar 6;11:199. doi: 10.3389/fgene.2020.00199 (PMC7069355; doi:10.3389/fgene.2020.00199)
Supplement: Supplementary file 7 [file Presentation_1.pdf]

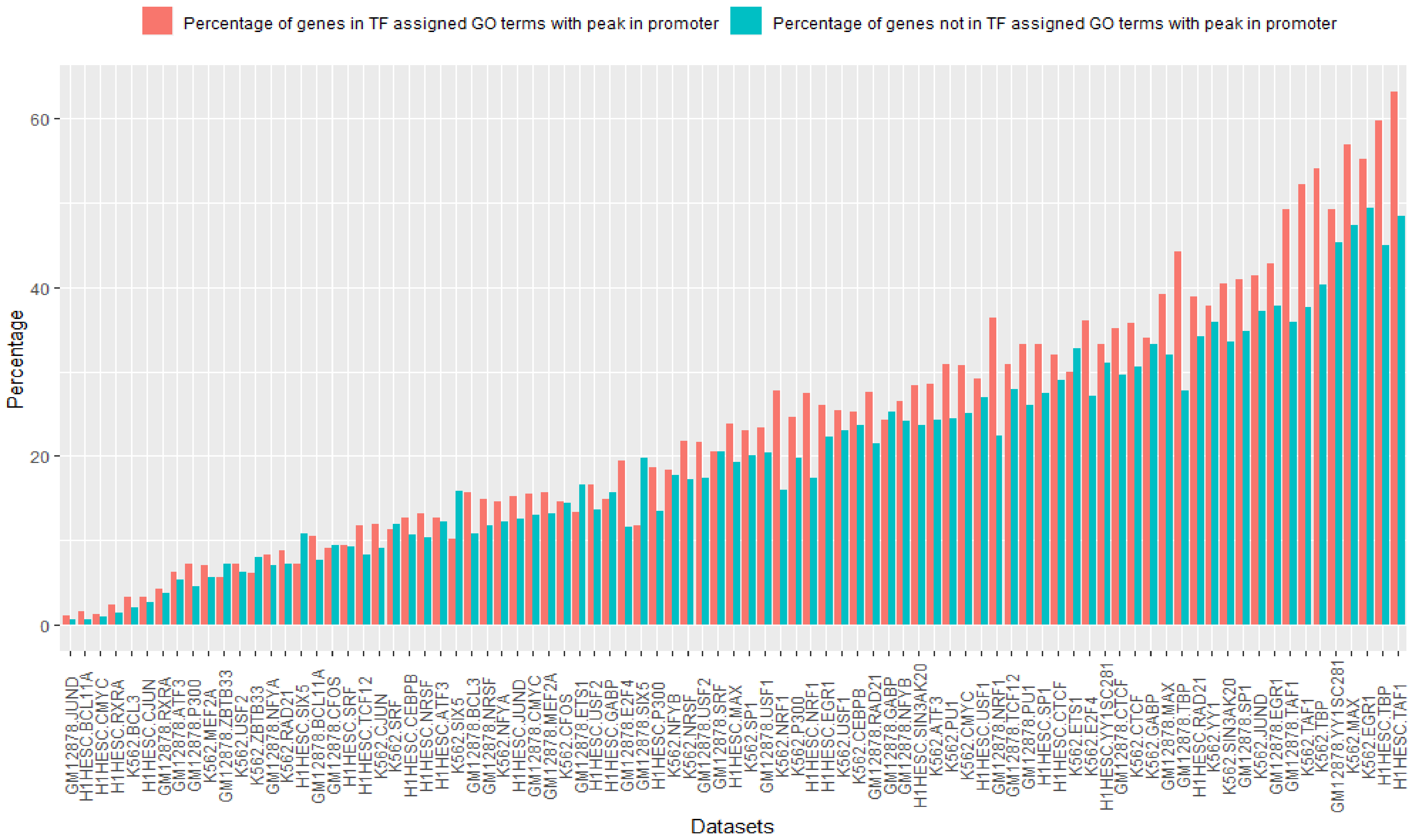

Supplementary Figure 1. To support our assumption that TFs tend to regulate genes in the biological processes to which they belong, we compared the percentage of genes that promoter covers with ChIP-Seq peak in the assigned GO BP terms and not in the assigned GO BP terms for TFs. The GO BP terms assignments for each TF were extracted from the human annotation Bioconductor package *org.Hs.eg.db.* Most of the ChIP-Seq data sets used in this paper show a higher percentage of genes in TF assigned GO terms with peak in promoter region than genes not in TF assigned GO terms. GO terms with less than 1000 genes were used in this figure. We also performed a proportion test for this, the results can be found in supplementary table 2.

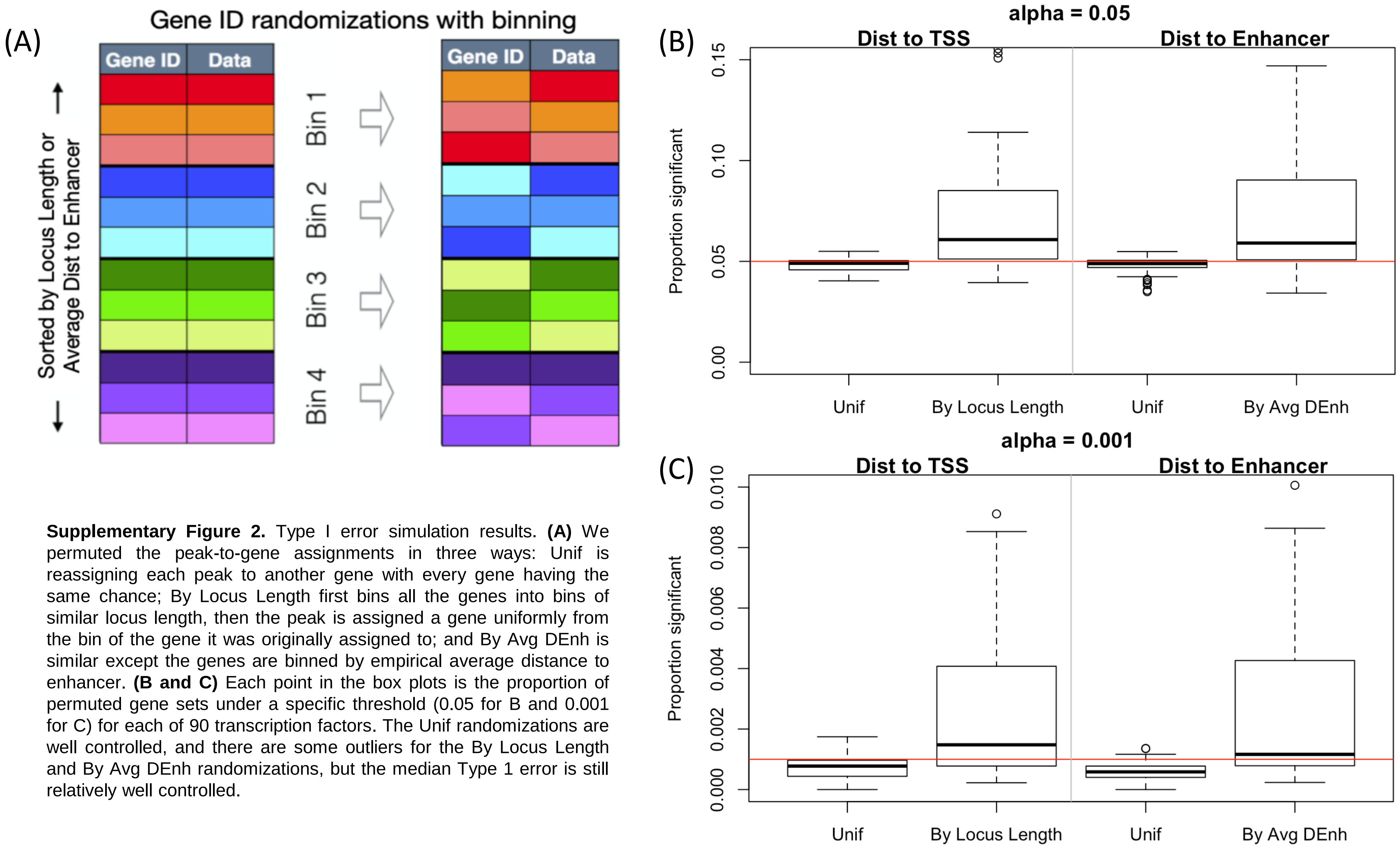

Supplementary Figure 2. Type I error simulation results. **(A)** We permuted the peak-to-gene assignments in three ways: Unif is reassigning each peak to another gene with every gene having the same chance; By Locus Length first bins all the genes into bins of similar locus length, then the peak is assigned a gene uniformly from the bin of the gene it was originally assigned to; and By Avg DEnh is similar except the genes are binned by empirical average distance to enhancer. **(B and C)** Each point in the box plots is the proportion of permuted gene sets under a specific threshold (0.05 for B and 0.001 for C) for each of 90 transcription factors. The Unif randomizations are well controlled, and there are some outliers for the By Locus Length and By Avg DEnh randomizations, but the median Type 1 error is still relatively well controlled.

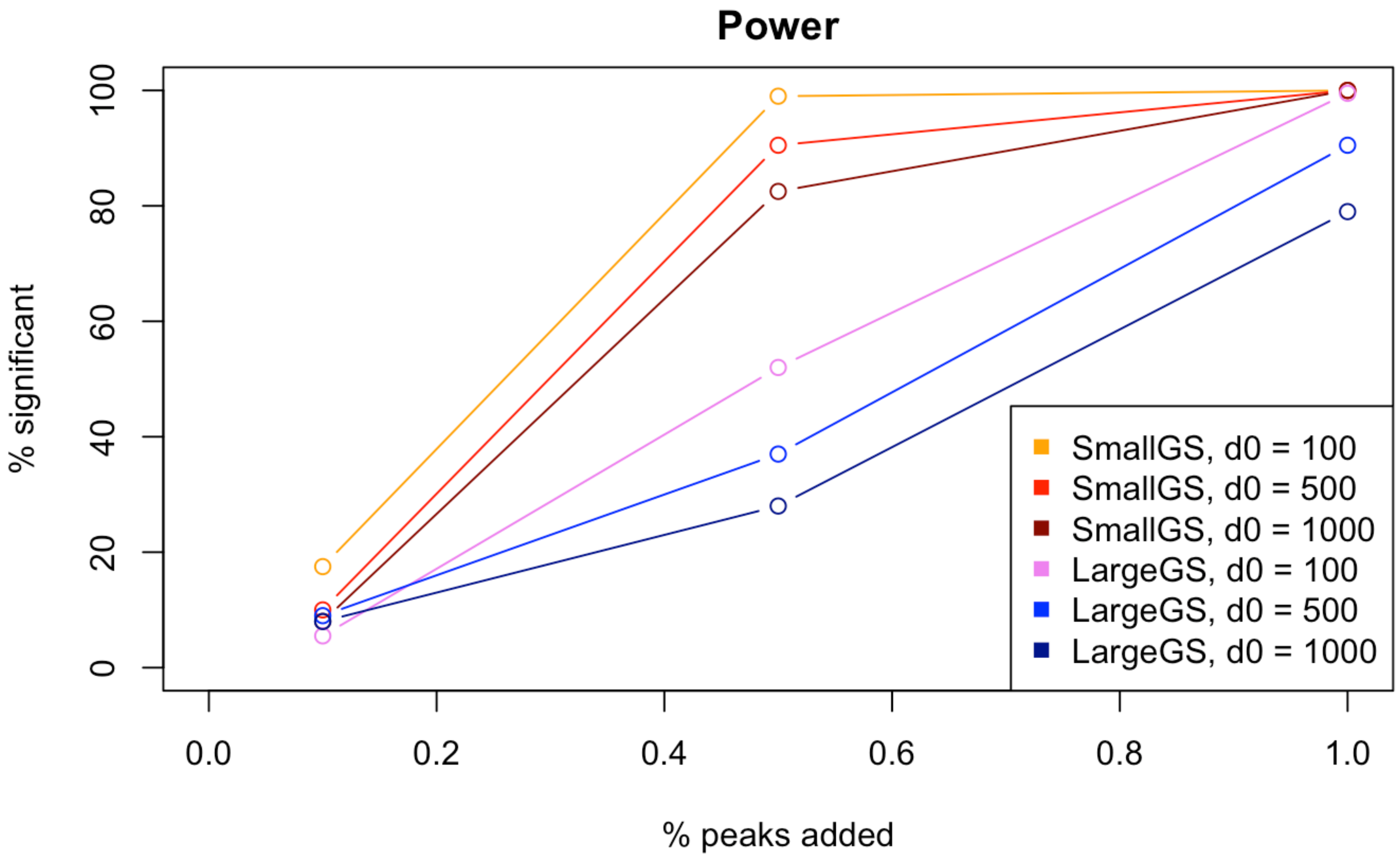

Supplementary Figure 3. Power simulation results. We generated pseudo-enrichment data by first starting with a permuted peak set in bins of locus length, and then added peaks to genes to a particular gene set. We chose a small (471 genes) and a large (1717 genes) gene set. The number of peaks added were 0.01%, 0.05%, or 0.1% of the total number of peaks in the experiment, which was 4839. Peak distances were added based on the following distribution: $P(Dist = x) = \exp\left(-\frac{x}{d0}\right)$ with d0 being the average distance from the TSS and the choices of d0 being 100,500, or 1000. As expected, when more peaks were added, peaks were added closer, or the smaller gene set was used (higher proportion of closer peaks in the gene set), the power increased.
